# A new phosphate-starvation response in fission yeast requires the endocytic function of myosin I

**DOI:** 10.1242/jcs.171314

**Published:** 2015-10-15

**Authors:** Edoardo Petrini, Victoire Baillet, Jake Cridge, Cassandra J. Hogan, Cindy Guillaume, Huiling Ke, Elisa Brandetti, Simon Walker, Hashem Koohy, Mikhail Spivakov, Patrick Varga-Weisz

**Affiliations:** 1 Nuclear Dynamics, Babraham Institute, Cambridge CB22 3AT, USA; 2 Imaging Facility, Babraham Institute, Cambridge CB22 3AT, USA

**Keywords:** Myosin, Phosphate sensing, Endocytosis

## Abstract

Endocytosis is essential for uptake of many substances into the cell, but how it links to nutritional signalling is poorly understood. Here, we show a new role for endocytosis in regulating the response to low phosphate in *Schizosaccharomyces pombe*. Loss of function of myosin I (Myo1), Sla2/End4 or Arp2, proteins involved in the early steps of endocytosis, led to increased proliferation in low-phosphate medium compared to controls. We show that once cells are deprived of phosphate they undergo a quiescence response that is dependent on the endocytic function of Myo1. Transcriptomic analysis revealed a wide perturbation of gene expression with induction of stress-regulated genes upon phosphate starvation in wild-type but not Δ*myo1* cells. Thus, endocytosis plays a pivotal role in mediating the cellular response to nutrients, bridging the external environment and internal molecular functions of the cell.

## Abstract (precis)

**Highlighted Article:** The endocytic machinery, including the type 1 myosin Myo1, is required to establish a quiescence response to low-phosphate stress in fission yeast.

## INTRODUCTION

Endocytosis involves proteins acting in membrane remodelling and force-generating actin filament assembly (Galletta and Cooper, 2009; Goode et al., 2015; Kovar et al., 2011). In *Schizosaccharomyces pombe* (*S. pombe*) myosin I (Myo1), one of the best-known components of the endocytic machinery, is present in only one isoform and is dispensable for survival (Lee et al., 2000; Toya et al., 2001). We exploited this characteristic to investigate the role of endocytosis in nutrient sensing and focused on phosphate sensing.

Imbalance in the intracellular levels of inorganic phosphate has important consequences on every aspect of the cell biology (Bergwitz and Jüppner, 2011). Studies in budding and fission yeast have identified the core machinery responsible for phosphate sensing, the signal transduction (PHO) pathway (Carter-O'Connell et al., 2012; Henry et al., 2011; Lenburg and O'Shea, 1996; Mouillon and Persson, 2006). Here, we show that the phosphate-starvation response requires endocytosis.

## RESULTS AND DISCUSSION

### Δ*myo1* cells are insensitive to phosphate starvation

**Fig. 1.**
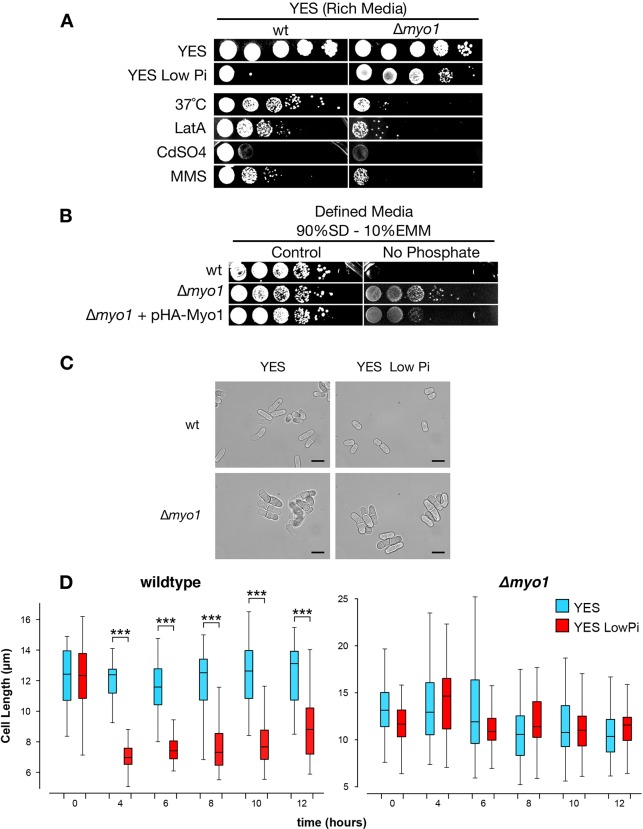
***Myo1*-deleted mutants exhibit no loss of growth in low-phosphate-containing medium.** (A) Serial dilution viability assay of wild-type (wt) and Δ*myo1* cells under stress conditions [YES LowPi, 0.5 µM LatA, 0.5 mM CdSO_4_, 0.01% (v/v) MMS]. (B) Serial dilution viability assay of Δ*myo1* and control cells in 90% SD, 10% EMM medium with and without phosphate. *HA–myo1* expression *in trans* was driven by the NMT1 promoter. Similar complementation results were obtained with a *myo1* expressed from an endogenous promoter (see Fig. 4A). (C) Brightfield images of wild-type or Δ*myo1* cells grown for 4 h in YES or low-phosphate YES. Scale bar: 10 µm. (D) Box-and-whisker plot showing length measurements of yeast grown for 4–12 h in YES or low-phosphate YES. The box represents the 25–75th percentiles, and the median is indicated. The whiskers show the 10–90th percentiles. The length of 50 cells for each condition was measured using the line tool of ImageJ. ^***^P<0.001 (Student's *t*-test).

We found that Myo1-deficient cells respond differently to various challenges compared to wild-type cells (Fig. 1A). Δ*myo1* cells were sensitive to the actin-depolymerising drug latrunculin A (LatA), treatment with cadmium sulphate, the DNA-damaging agent methylmethane sulfonate (MMS) and growth at elevated temperatures, revealing a general loss of fitness of the Δ*myo1* mutant under challenging conditions. By contrast, Δ*myo1* cells were unaffected by stress induced by low phosphate levels, as opposed to wild-type cells, which were severely affected by this condition (Fig. 1A,B).

### Phosphate starvation drives cells into a quiescent-like status

Under specific conditions, nutrient starvation drives fission yeast cells into a quiescent state, where they stop proliferating but maintain viability, and this response is linked to changes in cell shape (Yanagida, 2009). We found that wild-type cells in the low-phosphate condition appeared smaller and rounder compared to their counterpart grown in normal phosphate medium, and this was apparent as soon as 4 h (Fig. 1C,D). Δ*myo1* cells were misshapen and often multi-septated as described previously (Lee et al., 2000; Toya et al., 2001), but did not show substantial changes in shape in the low-phosphate medium (Fig. 1C,D).

**Fig. 2.**
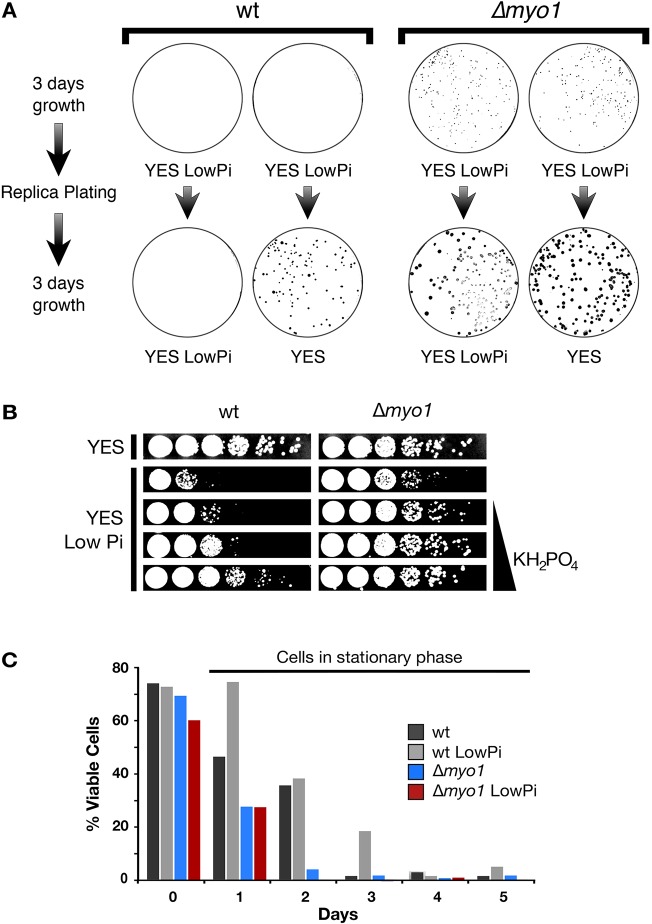
**Cells enter a quiescent-like state in the low-phosphate stress condition.** (A) ∼300 wild-type (wt) or Δ*myo1* cells were plated on low-phosphate YES. After 3 days, cells were replica plated onto either low-phosphate YES or YES (normal phosphate) media and incubated for an additional 3 days (shown here are representative results of three repeat experiments). (B) Serial dilution viability assay testing cellular growth of wild-type and Δ*myo1* strains in YES LowPi containing increasing amounts of phosphate (phosphate removed, 1 mM, 5 mM and 10 mM KH_2_PO_4_ added). Phosphate was removed from YES as described in the Materials and Methods and was re-constituted with defined amounts of phosphate using KH_2_PO_4_. Panels are also shown in Fig. 4A. (C) Cells were grown in YES or YES LowPi media for the indicated amount of time, then cell viability was measured as the percentage of colony-forming units on the total number of cells plated in YES. Two independent experiments have been carried out with similar outcomes; one representative experiment is shown.

When we tested growth from single cells, no wild-type colony was observed during phosphate starvation on solid medium; however, after replica-plating onto phosphate-containing medium, colonies became evident after a further 3 days' growth (Fig. 2A). In contrast, Δ*myo1* colonies were observed in all the conditions tested. Wild-type cell growth was directly proportional to phosphate concentration in the medium, but Myo1-deficient cells did not respond to changes in phosphate concentration (Fig. 2B).

Fission yeast (unlike budding yeast) lose viability over time after reaching stationary phase in rich media (Yanagida, 2009; Zuin et al., 2010), but no reduction in cell viability was observed in cells maintained in low-phosphate medium for the wild-type strain compared to cells maintained in high phosphate medium over several days (Fig. 2C). These cells retained viability better when compared to their counterpart grown in YES medium. In contrast, a rapid reduction of viability was detected in Δ*myo1* mutants both in low and normal phosphate conditions.

Thus, phosphate withdrawal drives cells into a quiescent-like status and entering this state is dependent on Myo1.

### Global transcriptomic changes in response to phosphate starvation are absent in Δ*myo1* cells

**Fig. 3.**
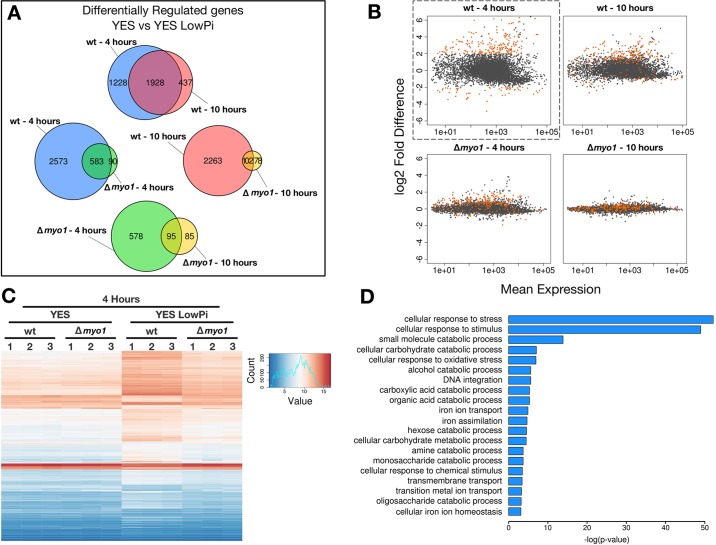
**Gene expression changes linked to the low-phosphate stress response are not triggered in Δ*myo1* cells.** (A) Venn diagrams showing the extent of overlap between differentially expressed genes (*P*<0.01) for each experimental condition. (B) Genes showing a log_2_ fold change above or below 2 and −2, respectively, in wild-type (wt) cells after 4 h of phosphate starvation are highlighted in orange (top-left scatterplot, dashed box). The same genes are then visualized in the remaining conditions. (C) Heatmap showing hierarchical clustering of the most differentially expressed genes at 4 h in all the comparisons (log_2_ fold change, <−2 or >2, *P*<0.01). (D) The identified genes were subjected to Gene Ontology analysis; negative log *P*-values are used to depict the most significant terms.

Previously characterised quiescence states show a profound alteration in gene expression patterns (Marguerat et al., 2012; Shimanuki et al., 2007; Wilhelm et al., 2008). We identified a large number (>3000) of differentially regulated genes by mRNA-Seq (mRNA isolation followed by next-generation sequencing) in wild-type cells at 4 and 10 h after switch to low-phosphate medium (Fig. S1A, at later time points there was outgrowth of suppressor mutants). A tight overlap was observed in wild-type cells when we compared the early (4 h) to late (10 h) response, suggesting that a core subset of genes is switched on and maintained during phosphate starvation (Fig. 3A). The majority of phosphate-starvation response genes were not triggered in absence of Myo1 (Fig. 3A).

We defined signature genes of this quiescence response by applying a magnitude threshold to the differentially regulated genes after 4 h phosphate starvation in wild-type cells (Table S1), tracking them in the remaining conditions (Fig. 3B). At the later time point, these signature genes remained responsive in wild-type cells, with most of them away from the zero line. However, in the Δ*myo1* mutant, most of these genes were not responding to the low-phosphate stress both at the early and late time points. Hierarchical clustering of the most responsive genes highlights the lack of the gene expression response of the Δ*myo1* mutant under low-phosphate conditions (Fig. 3C; Fig. S1B). Gene ontology analysis of the differentially regulated genes revealed that a significant proportion were involved in cellular response to stress and in different catabolic processes, confirming that this is mainly a stress response (Fig. 3D). In summary, phosphate starvation leads to a dramatic change in gene expression, which is abolished when *myo1* is deleted.

### The endocytic function of Myo1 is required for the low-phosphate-induced quiescence response

**Fig. 4.**
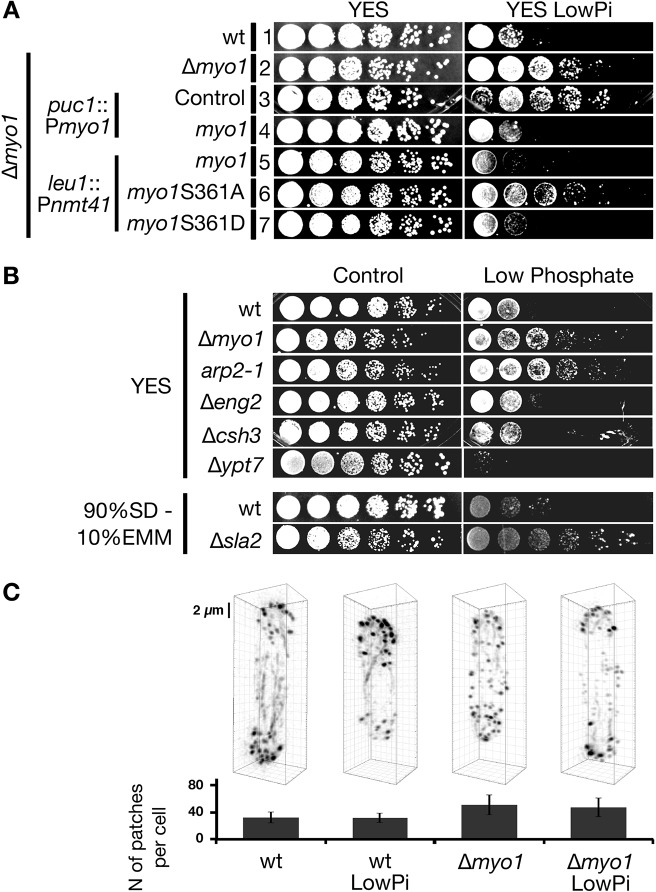
**Endocytic functions of Myo1 are required to trigger the stress response to low phosphate.** (A) Serial dilution viability assays of the rescue of the low-phosphate response phenotype of the Δ*myo1* strain [lane 1, wild type (wt); lane 2, Δ*myo1*; panels are replicated from Fig. 2B for comparison, the experiments shown in Figs 2B and 4A were performed in parallel] using stable integrants under the Myo1 native promoter (lane 3, Δ*myo1 puc1*::P*_myo1_*-*ura4*; lane 4, Δ*myo1 puc1*::P*_myo1_*-*myo1*) or under the *nmt41* promoter (lanes 5, Δ*myo1 leu1*::P*_nmt41_*-GFP-*myo1*; lane 6, Δ*myo1 leu1*::P*_nmt41_*-GFP-*myo1*S361A,; lane 7, Δ*myo1 leu1*::P*_nmt41_*-GFP-*myo1*S361D). (B) Serial dilution viability assays of cells defective in components required for endocytosis. (C) SIM images of phalloidin-stained actin in cells grown under the indicated conditions. Images were reconstructed in 3D and are shown rotated along the *y*-axis by 30°; representative images are shown. Scale bar: 2 µm. The graph underneath shows the average number of actin patches in each cell for each condition (mean±s.d.; *n*=50).

Myo1 plays a crucial role in endocytosis where it is required for vesicle internalization (Attanapola et al., 2009; Sirotkin et al., 2005, 2010); Myo1 is also required for the regulation of actin polymerization and patch formation at cortical sites (Evangelista et al., 2000; Lee et al., 2000), promoting polarised growth and sterol-rich membrane organization (Takeda and Chang, 2005). We took advantage of a point mutation in *myo1* Ser361 at the TEDS site, phosphorylation of which is specifically required for the endocytic function of Myo1 and its cellular localization, but which does not affect actin organisation inside cells (Attanapola et al., 2009). We tested growth in low phosphate of a Δ*myo1* strain complemented either with *myo1* carrying a S361A mutation, which cannot be phosphorylated, or a S361D mutation which acts as a phosphomimetic (Attanapola et al., 2009) (Fig. 4A). Ectopic expression of S361A *myo1* (without the TEDS phosphorylatable residue) was not able to rescue the Δ*myo1* phenotype (cells grew well in low phosphate) (Fig. 4A, Lane 6). Conversely, the phosphomimetic mutation S361D *myo1* rescued the loss of wild-type *myo1*, restoring sensitivity to low-phosphate stress to the same extent as full-length *myo1* (Fig. 4A, compare lane 7 with lanes 1, 4, 5). Thus, the role of Myo1 in endocytosis appears to be specifically required for the response to low-phosphate stress.

We asked whether other components of the endocytic machinery were required for the response to low-phosphate stress (Fig. 4B). The temperature-sensitive mutant *arp2-1* fails to form a functional Arp2/3 complex (Morrell et al., 1999), which is directly activated by Myo1 to stimulate actin nucleation and branching at sites of endocytosis (Lee et al., 2000; Sirotkin et al., 2005). Sla2/End4 is an adaptor protein recruited very early in the endocytic process, immediately after clathrin appearance, and it is essential for endocytosis (Iwaki et al., 2004; Sirotkin et al., 2010). To verify the involvement of the later steps of endocytosis, we tested deletion mutants of *eng2* and *csh3*, which form a newly discovered endocytic module (Encinar del Dedo et al., 2014), and Ypt7, a protein involved in the fusion of endocytic vesicles with vacuoles (Bone et al., 1998; Murray and Johnson, 2001).

We observed that the *arp2-1* mutation and *sla2/end4* deletion conferred resistance to low-phosphate stress to a similar extent as *myo1* deletion (Fig. 4B). Mutations in *ypt7*, *eng2* or *csh3* did not lead to increased proliferation in YES LowPi compared to controls (Fig. 4B).

To verify that actin dynamics were not altered during phosphate starvation, we monitored changes in actin patch formation and localization using structural illumination (SIM) microscopy in order to obtain an accurate estimate of the average patch concentration (Fig. 4C). Consistent with previous observations (Lee et al., 2000), we found an increase in actin patches between wild-type cells and the Δ*myo1* mutant. No difference could be observed between the normal and low-phosphate condition, both in actin patches numbers and localization along the cell body.

Given that cytoskeletal proteins myosin I, actin and ARP2 have been shown to have nuclear functions in gene expression in mammalian cells (Percipalle et al., 2006; Pestic-Dragovich et al., 2000; Philimonenko et al., 2004; Ye et al., 2008), we tested whether Myo1 in fission yeast was found in the nucleus and associated with chromatin but did not find any evidence for that (Fig. S2A,B).

We show that the impairment of endocytosis has important consequences on the capability of cells in responding to phosphate starvation, suggesting that internalisation is required to sense the lack of phosphate or to transduce this signal inside the cell in order to appropriately respond to the lack of this essential nutrient. We favour the idea that endocytosis is required for sensing rather signalling low phosphate, given that mutating early components of endocytosis, like Arp2 and Sla2/End4, abrogated the phosphate starvation response.

We propose that the isolation and intake of the external medium into vesicles is required to place cytoplasmic phosphate reservoirs and the extracellular environment in close proximity and this is required to sense phosphate gradients effectively, e.g. by comparing with the cytoplasmic phosphate levels. Our work is, to our knowledge, the first description that links nutrient sensing to endocytosis and cytoskeletal protein function. Future studies should address whether the endocytic machinery is also required for nutrient sensing and coordinated responses to different stress in multicellular organisms.

## MATERIALS AND METHODS

### Yeast culture

Cells were grown at 30°C unless otherwise stated. Yeast extract supplemented (YES, pH 5.5) medium was used for normal phosphate conditions; low-phosphate medium (YES LowPi) was obtained as described previously (Schweingruber and Schweingruber, 1981). Briefly, MgSO_4_ was added to 10 mM to YES media, then NH_4_OH was added dropwise until a visible precipitate formed, which was removed using a 0.02 µm filter, the pH was adjusted with HCl to 5.5 and the medium was autoclaved. KH_2_PO_4_ was added back to re-supplement phosphate to YES in Fig. 2B, YES LowPi+1 mM KH_2_PO_4_, pH.5.5; YES LowPi+5 mM KH_2_PO_4_, pH.5.3, YES LowPi+10 mM KH_2_PO_4_, pH.5.2. 90% SD, 10% EMM was used as defined medium, with 10 mM KH_2_PO_4_ or no phosphate added (Henry et al., 2011).

The strains used in this study are listed in Table S2.

### Growth assays

Mid-log phase cells were resuspended to a concentration of 7.5×10^6^ cells/ml, then fivefold serial dilutions were performed. Cells were transferred onto Petri dishes using a replicator, and incubated for 3 days at 30°C, unless otherwise stated.

### Viability measures

Mid-log phase cells were diluted to an optical density at 595 nm (OD_595_)=0.05 in YES or YES LowPi and grown at 30°C for 5 days. Every day, ∼300 cells were plated in solid YES media in triplicate. Plates were incubated for 3 days and subsequently photographed. Numbers of colonies on each plate were scored using FIJI image software.

### Phalloidin staining

Cells were fixed with 1% methanol-free formaldehyde, 60 min at 30°C in YES media. Samples were permeabilized with 1% Triton X-100, and stained with 2.2 µM Alexa-Fluor-555–Phalloidin (Life Technologies) for 60 min at 24°C.

### Microscopy and image analysis

Brightfield images for Fig. 1C,D were taken with an Olympus BX61 microscope, 100× objective and the Cell^F^ imaging software. Images for Fig. 4C were obtained with a Nikon N-SIM (Structural Illumination Microscopy) system, using a 100×1.49 NA oil-immersion objective. Images were acquired using the 3D reconstruction algorithm embedded in the NIS Elements software package (Nikon). Image analysis was performed using Imaris (Bitplane) to identify and measure the number of actin patches present in each cell.

### mRNA-Seq and ChIP-Seq library preparation

Cells were diluted in 200 ml of either YES or YES LowPi to a density of 0.05 OD_595_. Cultures were incubated at 30°C, 5×10^7^ cells were taken after 4 and 10 h. RNA extraction was performed with hot phenol (Köhrer and Domdey, 1991); mRNA isolation and library preparation were performed with the NEBNext^®^ Ultra™ RNA Library Prep Kit. Multiplexing was performed with the iPCR system (Quail et al., 2012). We performed 50-bp paired-end sequencing using a HiSeq 2500. The protocol of ChIP-seq is available on request.

### Sequencing data analysis

Raw sequencing data were aligned against the *S. pombe* genome (ASM294v2) with TopHat v.2.0.12 (Trapnell et al., 2009) using default settings. Initial data processing was performed with SeqMonk (http://www.bioinformatics.babraham.ac.uk/projects/seqmonk/) “DESeq2” v1.6.3 (Love et al., 2014) was used for differential gene expression analysis, defining the level of significance at *P*<0.01. BinGO (Maere et al., 2005) was used for Gene Ontology analysis, using the Hypergeometric Test for overrepresentation, with multiple testing correction (Benjamini-Hochberg).

### Accession code

The RNA-Seq and ChIP-Seq Gene Expression Omnibus (GEO) accession code is GSE67126.
